# Potential of using an engineered indole lactic acid producing *Escherichia coli* Nissle 1917 in a murine model of colitis

**DOI:** 10.1038/s41598-024-68412-9

**Published:** 2024-07-30

**Authors:** Chrysoula Dimopoulou, Priscila Regina Guerra, Martin Steen Mortensen, Katja Ann Kristensen, Mikael Pedersen, Martin Iain Bahl, Morten Alexander Otto Sommer, Tine Rask Licht, Martin Frederik Laursen

**Affiliations:** 1 National Food Institute, Technical University of Denmark, Kgs. Lyngby, Denmark; 2 The Novo Nordisk Foundation Center for Biosustainability, Technical University of Denmark, Kgs. Lyngby, Denmark

**Keywords:** Biochemistry, Biotechnology, Microbiology, Molecular biology, Biomarkers, Diseases, Gastroenterology, Pathogenesis, Engineering

## Abstract

The gut microbiome is a significant factor in the pathophysiology of ulcerative colitis (UC), prompting investigations into the use of probiotic therapies to counter gastrointestinal inflammation. However, while much attention has been given to the therapeutic potential of microbes at the species and strain level, the discovery and application of their metabolic products may offer more precise and controlled solutions in battling disease. In this work, we examined the therapeutic potential of indole lactic acid (ILA) to alleviate inflammation in a murine model of colitis. A previously constructed ILA-producing *Escherichia coli* Nissle 1917 strain (EcN *aldh*) and its isogenic non-ILA producing counterpart (EcN) were studied in a murine model of Dextran Sodium Sulfate (DSS) induced colitis. The colitic animals suffered from severe colitic symptoms, with no differentiation between the groups in body weight loss and disease activity index. However, three days after cessation of DSS treatment the EcN *aldh*–treated mice showed signs of reduced intestinal inflammation, as manifested by lower concentrations of fecal lipocalin-2. Additionally, expression analysis of the inflamed tissue revealed distinct effects of the EcN *aldh* strain on proteins associated with intestinal health, such as TFF3, occludin and IL-1β expression. These results show no impact of EcN or EcN *aldh* on acute DSS-induced colitis, but suggest that in particular EcN *aldh* may assist recovery from intestinal inflammation.

## Introduction

Ulcerative colitis (UC) is one of the principal types of inflammatory bowel disease (IBD), a chronic condition that manifests as recurrent mucosal inflammation in the colon and rectum^1^. Its etiology is not clearly determined. Genetic background and mucosal immune dysregulation play central roles, in concert with a number of environmental factors, with the gut microbiota being a key factor^2^. Current first-line therapies involve anti-inflammatory drugs, such as corticosteroids, and in cases progressing from mild-to-severe disease, biological agents that intricately target immunological actors such as TNF or integrin α_4_β_7_ are commonly used^3^. However, a clinical response is not achieved in all patients and their prolonged use may cause significant adverse effects^3–5^. Hence, there is a need for alternative modalities with high clinical efficiency, fewer side effects and prolonged remission periods.

Gut bacterial species and their metabolites have been associated with disease outcomes^6,7^. Thus, alternative therapies, based on gut microbiome manipulations, are also being explored. Microbiota therapies against UC have shown promise towards disease cessation and remission^8^, although clinical studies on probiotics and/or fecal microbiome transplantation (FMT) treatments exhibit high variability in remission rates^9,10^. Since the exact molecular mechanisms of action of probiotics and FMTs are still unknown, it is difficult to standardize the therapies and develop reproducible interventions.

One solution is to combine knowledge on gut metabolites and synthetic biology to engineer advanced microbial therapeutics (AMTs) that can colonize the gastrointestinal tract and express specific bacterial metabolites *in situ*^11^. Localized delivery minimizes pharmacological loss of the active compound and reduces systemic exposure. Furthermore, engineered probiotic strains, such as *Escherichia coli* Nissle 1917 (EcN), allow for the development of complex genetic circuits that control strain colonization^12^, regulate the expression of specific metabolites^13^ and enable biocontainment of the strains^14^. This makes AMTs promising alternatives to both conventional therapies and traditional probiotic approaches. Especially since natural producers are typically not efficient colonizers of the colon^15,16^ and the production of the respective metabolites cannot be tightly controlled and, subsequently, lead to standardized therapies, as it is dependent on the composition of the microbiome^17^.

Several studies have used AMTs to alleviate colitis symptoms^18–20^, but so far none has employed microbial metabolites. Since such metabolites inherently interact with the intestine, their use as therapeutic agents may improve therapeutic specificity and potency with marginal side effects^21^. One promising candidate is indole lactic acid (ILA). Cumulative evidence suggests that ILA and ILA-producing species protect the intestinal barrier and decrease inflammation^22–25^. In addition, EcN has been reported to be a potent anti-colitic agent, whose properties are comparable to the golden standard of UC treatment, mesalazine^26,27^. EcN is known to interact with both the gut microbiota and the host, leading to its intestinal inflammation amelioration properties. Mechanisms of actions include direct and indirect antagonistic effects against enteroinvasive pathogens, reinforcement of the gut barrier, and immune-modulatory and anti-inflammatory effects^28,29^.

Previously, we developed an engineered EcN, namely EcN *aldh*, which boosts ILA production in the gut microbiome^30^. In the present study, we investigated the therapeutic potential of EcN *aldh* against DSS-induced colitis and epithelial recovery in a preclinical mouse model. We present an AMT that, upon colonization, increases systemic ILA, but does not alter acute disease progression. However, three days after cessation of DSS-treatment, EcN *aldh* mice showed lower intestinal inflammation levels and improvement in gene expression markers of intestinal epithelial health suggested an enhanced healing process in the presence of the AMT.

## Results

### Colitic symptoms of the DSS-treated mice

**Figure 1:**
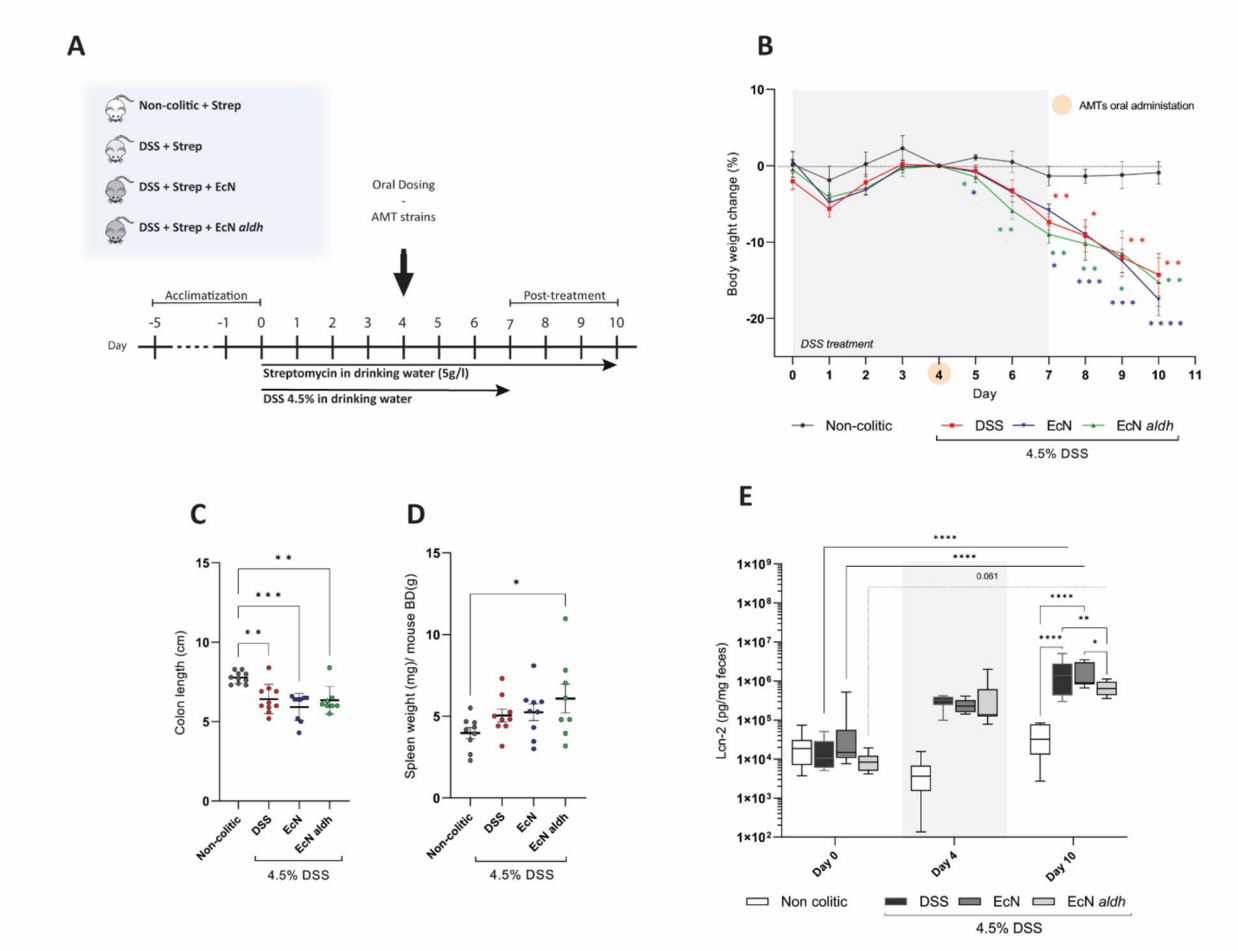
DSS-induced colitis: macroscopic observations and markers of disease. (**A**) Experimental setup. (**B**) Body weight loss calculated relatively to the AMT administration day (Day 4). Statistical analysis performed with the mixed effects model (REML), uncorrected Fisher’s LSD. (**C**) Colon length measurements and (**D**) spleen weight measurements on termination day. Statistical significance was calculated by Kruskal Wallis, uncorrected Dunn test, and by ordinary one-way ANOVA, Fisher’s LSD test, respectively. (**E**) Fecal lcn-2 measurements on selective days of the experiment. Statistical analysis was performed with a two-way ANOVA, Tukey correction (adjusted p-values are depicted on the illustration). All data is shown as the mean ± SEM of the biological replicates from each group (n = 10, 10, 9, 8 in the non-colitic, DSS, EcN and EcN *aldh* groups respectively). *p < 0.05, **p < 0.01, ***p < 0.001, ****p < 0.0001.

The study comprised seven days of DSS treatment with orally administered AMTs on Day 4 and a post-treatment period of three days (Fig. 1A). All DSS-treated animals exhibited similar symptoms, with drastic weight loss occurring from Day 5 onwards (Fig. 1B). The severity of the colitis symptoms was further displayed by the Disease Activity Index calculations, with no significant differences between the DSS treated groups (Supplementary Fig. S1). Colon length measurements indicated similar disease severity among the colitic mice groups, with the non-colitic colons being significantly longer (Fig. 1C). All colitic mice had slightly enlarged spleens, compared to the untreated group, only reaching statistical significance in the EcN *aldh* group (Fig. 1D).

Lipocalin-2 (lcn-2), an acute-phase protein of the innate immune response, exhibits bacteriostatic activity^31^ and is also involved in the regulation of systemic inflammation^32,33^ and mucosal repair^34^. Its expression is increased distinctly in inflammatory diseases^35–37^, and it is considered a great biomarker for disease progression. Fecal lcn-2, in particular, is an excellent real-time biomarker of intestinal inflammation considering its stability, sensitivity, and broad dynamic range to detect low-grade but also severe colitic symptoms^38,39^. Fecal lcn-2 was increased in all colitic groups by Day 10 as compared to the Day 0 fecal concentration (Fig. 1E). While no significant differences were detected on Day 0 among the groups (Kruskal Wallis, uncorrected Dunn test), on Day 10 administration of EcN *aldh* resulted in significantly lower levels of fecal lcn-2 compared to the other colitic groups (Fig. 1E), suggesting a beneficial effect of the strain on intestinal inflammation. The DSS and EcN groups displayed approximately a 40-fold increase compared to the control group (*p* < 0.00001, both comparisons), while EcN *aldh* exhibited a less pronounced tenfold increase in fecal lcn-2, which did not reach statistical significance (p = 0.118). The relatively high fecal lcn-2 levels observed in the control group is likely attributed to the mild inflammation resulting from streptomycin administration^40^. Serum lcn-2 as a general marker of pathophysiological conditions^41^, did not differ among the groups, although all groups experienced an increase by Day 9 (Supplementary Fig. S1B).

### Histological analysis of colonic tissue

**Figure 2:**
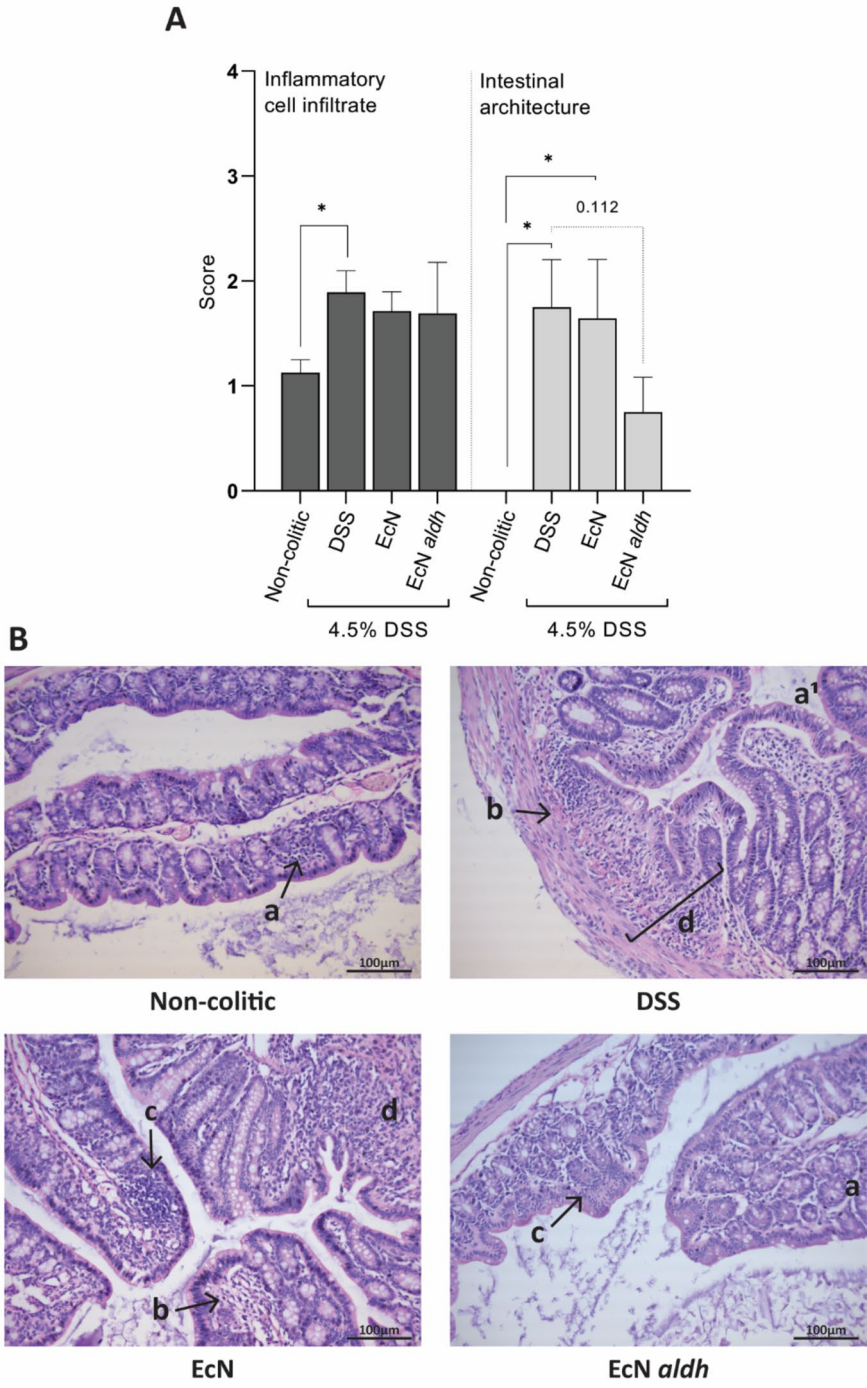
Histopathological analysis: influence of EcN *aldh* on inflammatory cell infiltrate and intestinal architecture. (**A**) Histological scores of colon sections. Data was generated by the histopathological analysis of 7 mice from each colitic group and 4 from the non-colitic group. The plots depict the mean ± SEM in each group. Statistical significance was calculated by Kruskal–Wallis, uncorrected Dunn’s test (inflammatory cell filtrate scores) and one-way ANOVA, uncorrected Dunn’s test (intestinal architecture scores). *p < 0.05 (**B**) Representative images of H&E-stained colon sections. Histological features: a: mucosal immune cell infiltration, a^1^: mucosal and submucosal immune cell infiltration, b: goblet cells loss, c: focal hyperplasia, d: ulcer formation.

The histopathological damage scores suggest no effect of the engineered strains on the inflammatory cell infiltrate compared to the DSS group, and a significant increase of the infiltrate in the DSS-treated mice compared to the non-colitic mice (Fig. 2A, left panel). DSS and EcN groups showed significantly higher scores in epithelial and mucosal damages compared to the non-colitic group, while this was not the case for the EcN *aldh* group, which scored lower than DSS and EcN (Fig. 2A, right panel), suggesting a slight impact of the strain. It should be noted that the non-colitic mice showed signs of mild inflammation, a known effect of streptomycin administration^40^, but no signs of architectural damage were observed in this group (Fig. 2A,B).

No major differentiation in the type of artifacts was observed among the groups (Fig. 2B). We detected inactive ulcers, focal hyperplasia, depletion of goblet cells and the presence of immune cell infiltrates that occasionally reached the submucosa in all colitic tissues processed, confirming that the mice suffered from moderate to severe symptoms. The presence of granulation tissue and process of restitution of cell lining suggest that the intestinal epithelium of the three colitic groups were in process of healing by Day 10. Overall, mice treated with EcN *aldh* showed a lower percentage of tissue architectural damages per histological cut (data not shown).

### AMTs and their impact on the gut metabolome

AMT colonization was similar for both strains with approx. 10^8^ and 10^9^ CFUs/g of gut content in the cecum and colon, respectively (Supplementary Fig. S2A). Antibiotic plates that selected only for the EcN and not the EcN *aldh* strain, confirmed that no substantial plasmid loss took place during the experiment (Supplementary Fig. S2B).

As EcN *aldh* influences the concentration of all three aromatic lactic acids (aLAs)^30^, besides ILA, we measured the concentration of phenyllactic acid (PLA) and 4-hydroxy-phenyllactic acid (4HPLA) in fecal samples from the mice. Before treatment, the average concentration of ILA was 1 ± 0.5 nmol/g, PLA 124.7 ± 30.2 nmol/g and 4HPLA 5 ± 0.9 nmol/g in the fecal samples. Introduction of streptomycin and DSS caused an increased ILA and 4HPLA and a decreased PLA concentration (REML model, Tukey test correction analysis, data not shown). When compared to the non-colitic and DSS animals, the AMTs appeared to only slightly affect the absolute values of the aLAs and no significant differences were observed between EcN and EcN *aldh* groups (Supplementary Fig. S3A-C).

**Figure 3:**
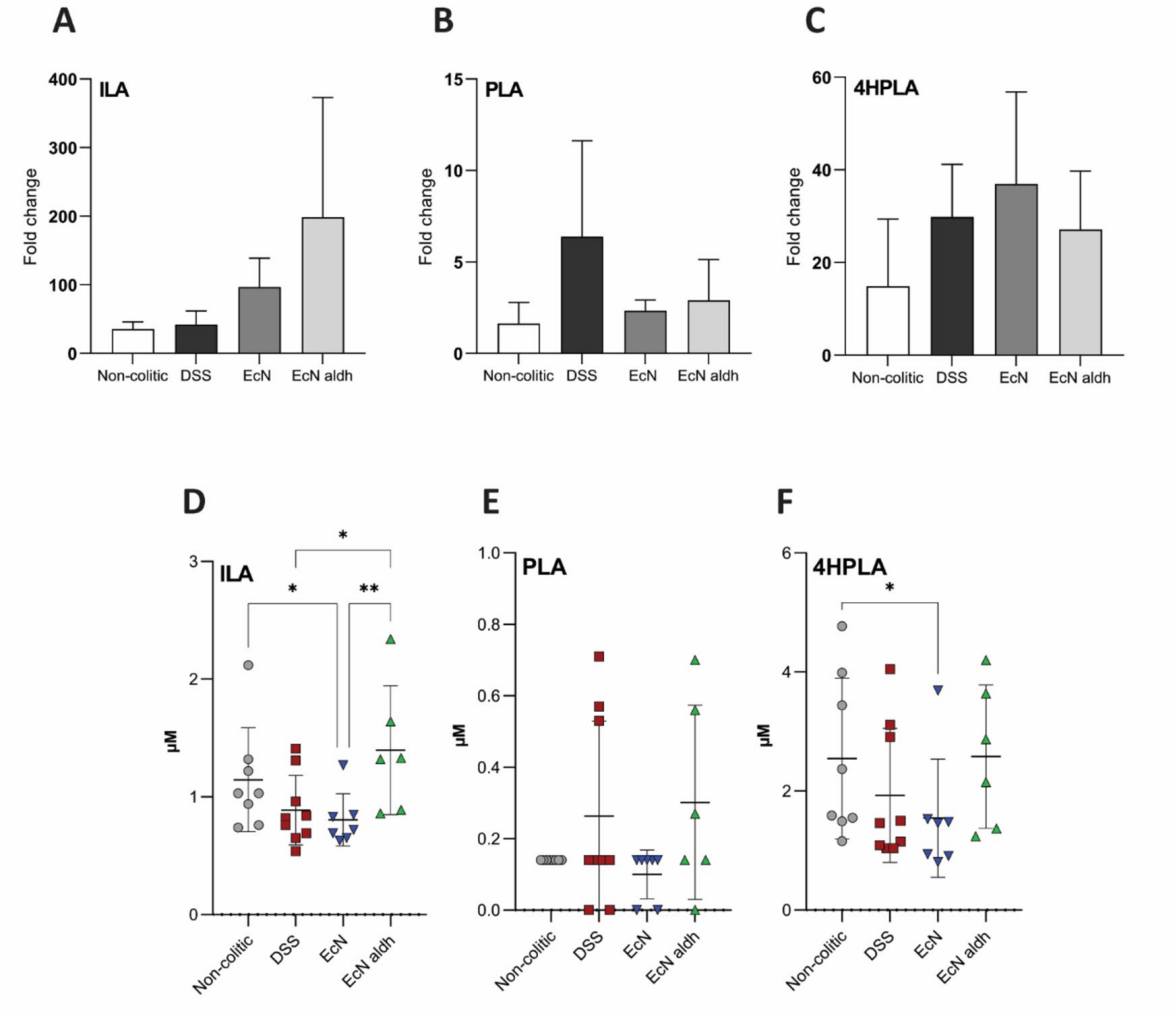
Impact of the AMT strains on fecal and serum aLAs. Fold change of (**A**) ILA, (**B**) PLA and (**C**) 4HPLA concentration in the fecal samples from D0 to D10. Values were calculated for each mouse individually. The bars depict the mean ± SEM of each group (n = 7, 9, 6, 4 in the non-colitic, DSS, EcN and EcN *aldh* groups respectively). Statistical significance was calculated with the Kruskal–Wallis method, uncorrected Dunn’s test. (**D**) ILA, (**E**) PLA and (**F**) 4HPLA concentrations in serum samples on day 9. The graphs illustrate the mean ± SD of the biological replicates (n = 8, 9, 7, 6 in the non-colitic, DSS, EcN and EcN *aldh* groups respectively). Each symbol represents an individual mouse. Statistical significance was calculated with the Kruskal–Wallis method, uncorrected Dunn’s test. *p < 0.05, **p < 0.01, ***p < 0.001.

To account for any individualized differences that may influence aLA production, we normalized the daily measurements for each mouse by their Day 0 concentration. AMTs increased ILA and 4HPLA concentrations, with higher ILA increase in EcN *aldh* and higher 4HPLA increase in EcN (Fig. 3A,C). However, none of these comparisons reached statistical significance. PLA concentrations did not increase by AMT administration (Fig. 3B). These results should however be interpreted with caution, since the fecal samples that were collected on the last day were too few to be considered a representative sample (Supplementary Table S1). The aAAs concentration decreased during DSS administration and increased during recovery (Supplementary Fig. S4A-C).

### EcN *aldh* affects the serum ILA levels

Higher serum ILA concentrations were observed in the EcN *aldh* treated mice on Day 9, its concentration was 1.6- and 1.7-fold higher in the EcN *aldh* than in the DSS and EcN group respectively (Fig. 3D). Although serum PLA was only detected in traces, the EcN *aldh* mice trended towards higher serum concentrations in comparison to the EcN mice (*p* = 0.08) (Fig. 3E). Similarly, serum 4HPLA exhibited a trend towards higher concentration in the EcN *aldh* mice when compared to the EcN (*p* = 0.08) (Fig. 3F). Analysis of serum samples on day 3 showed no previous differences among the groups (Supplementary Fig. S5A). Serum concentrations of tryptophan and phenylalanine on Day 9 appears elevated in the EcN *aldh* mice, although the effect seems to be driven by a single mouse (Supplementary Fig. S5B). No differences in serum tyrosine concentration were observed among the colitic animals (Supplementary Fig. S5B).

### AMTs influences expression of intestinal epithelial health markers

**Figure 4:**
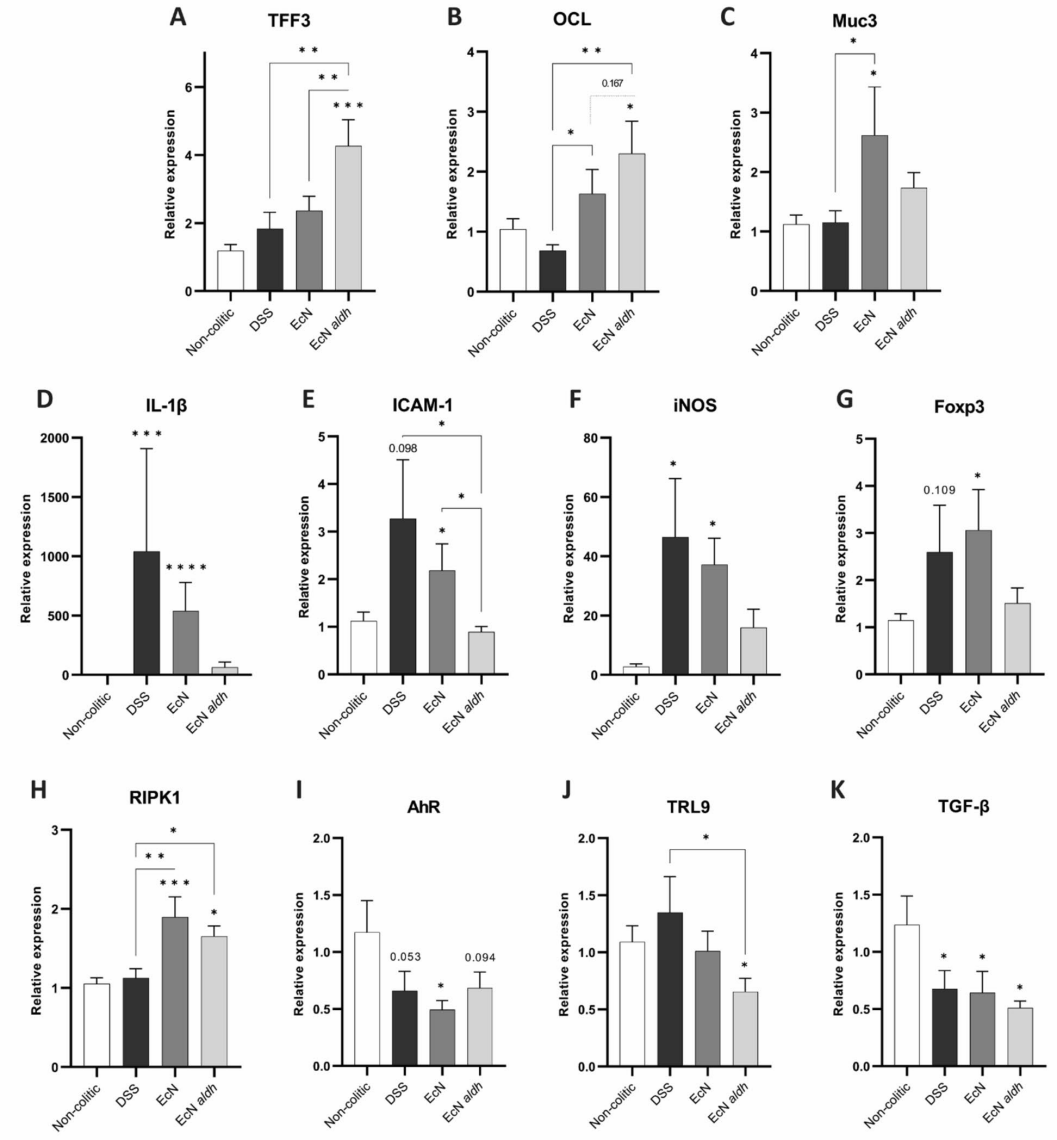
Impact of EcN *aldh* on molecular markers of intestinal epithelial recovery and inflammation. Relative expression of proteins related to (**A**–**C**) intestinal epithelial health (TFF3, OCL, Muc3), and (**D**–**H**) immunological markers (IL-1β, iCAM, iNOS, RIPK1, Foxp3, AhR, TRL9, TGF-β) in colonic tissue. The RT-qPCR results were normalized to the expression of NONO. The gene expression is shown relative to the non-colitic animals. Bars represent the mean ± SEM of the biological replicates (n = 9, 7, 7, 7; non-colitic, DSS, EcN and EcN *aldh* groups respectively). Statistical analysis was performed with one-way ANOVA, Fisher’s LSD test, except for IL-1β, iCAM-1, iNOS and TLR9 data that was Kruskal–Wallis, uncorrected Dunn’s test was used. Asterisks (*) on top of the bars note the p values in comparison to the non-colitic mice. *p < 0.05, **p < 0.01, ***p < 0.001, ****p < 0.0001.

The impact of AMTs was further examined by relative gene expression analysis on colonic tissue from the mice from the last day of the experiment and several effects on gene expression were observed (Fig. 4). Compared to the DSS and EcN, EcN *aldh* markedly upregulated Trefoil factor 3 (TFF3), a peptide with anti-apoptotic properties that is essential for epithelial repair^42^, suggesting a direct effect of ILA on TFF3 expression (Fig. 4A). Occludin (OCL), a protein integral to tight junction stability^43^ and gut barrier function^44^ was upregulated in both groups that received the AMTs, compared to DSS, although the effect was larger in the EcN *aldh* group (Fig. 4B). Expression of mucin 3 (Muc3), a glycoprotein responsible for epithelial restitution^45^, was mainly upregulated in EcN (Fig. 4C).

Analysis of mRNA expression of markers of intestinal inflammation revealed an effect of EcN *aldh* on the inflammatory response as well. IL-1β, a pro-inflammatory cytokine with essential roles in colonic inflammation^46^, was significantly upregulated in the DSS and EcN groups, with mean fold change of 1041 and 540 respectively, while EcN *aldh* mice exhibited a mean fold change of 63 when compared to the non-colitic group (Fig. 4D). Similarly, the intercellular adhesion molecule 1 (ICAM-1) expression, a molecule whose upregulation strongly correlates with increased DSS-colitis symptoms^47,48^, was significantly increased in the DSS and EcN groups, compared to the EcN *aldh* group (Fig. 4E). The expression of the inducible nitric oxide synthase (iNOS), an enzyme linked to aggravated inflammation due to excessive production of NO^49^ was also significantly upregulated only in the DSS and EcN groups (Fig. 4F). A similar trend was observed in Foxp3 expression, an anti-inflammatory protein when located in the colonic epithelium^50^, although the results were not statistically significant (Fig. 4G).

The receptor-interacting serine/threonine-protein kinase 1 (RIPK1), a protein integral to cell death and survival^51,52^, was significantly upregulated in both AMT-receiving groups compared to both non-colitic and DSS groups (Fig. 4H). The Aryl hydrocarbon Receptor (AhR), toll-like receptor 9 (TLR-9) and Tumor Growth Factor-beta (TGF-b) mRNAs had downregulation trends compared to the non-colitic group (Fig. 4I, J, K). All are implicated in immunological protection pathways in the gastrointestinal lumen^53–55^, and only TLR9 was significantly downregulated in the EcN *aldh* group, compared to the EcN group.

### Effect of the AMTs on bacterial diversity and community composition

**Figure 5:**
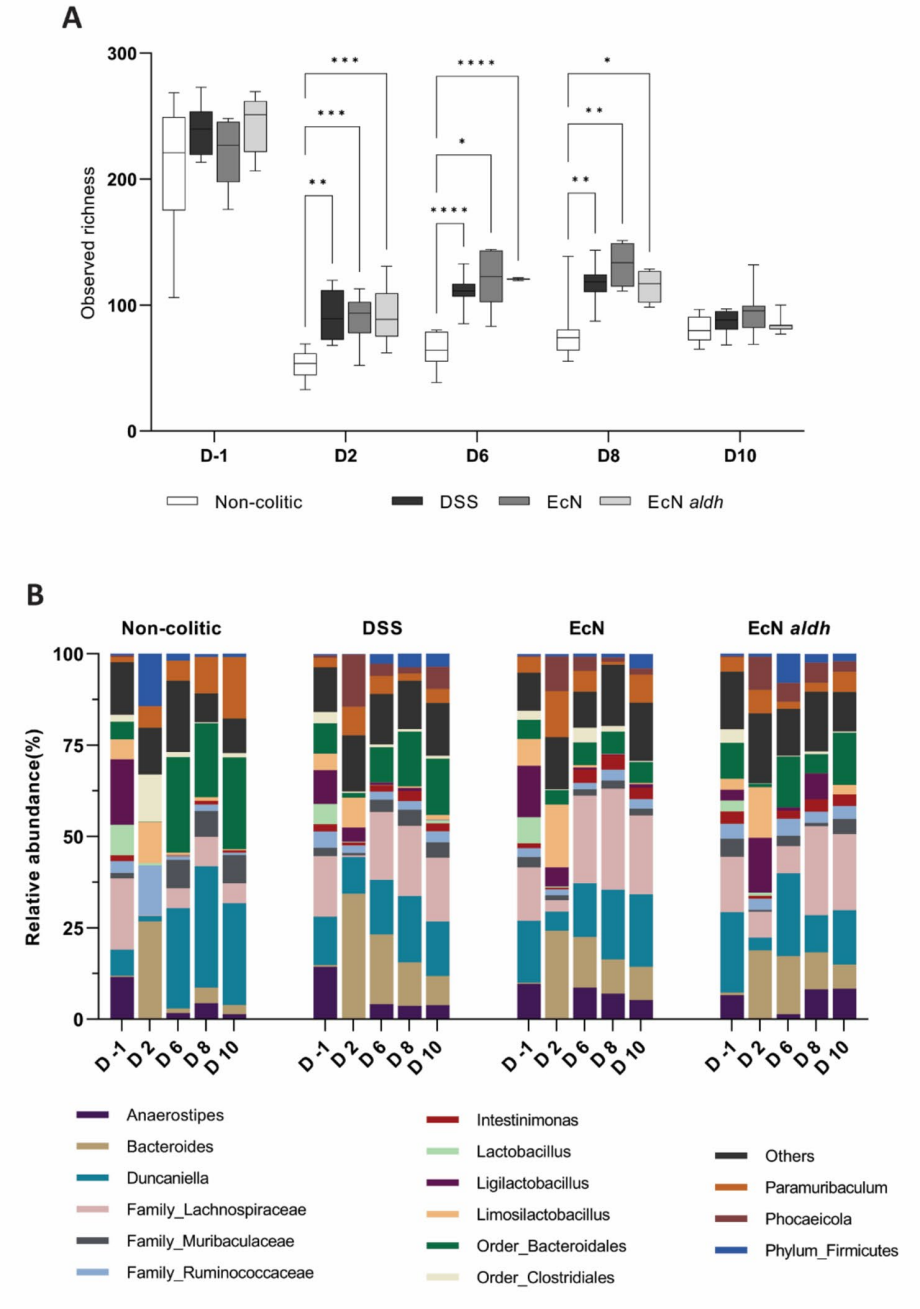
Compositional changes resulting from the AMT administration. (**A**) Observed richness of the four experimental groups. Box plots depict the median, minimum, and maximum values from the independent biological replicates on the selected days. (**B**) The microbiota composition of the experimental groups based on the detected genera on selected days of the experiment. (Relative abundance cutoff 0.1%) *q < 0.05, **q < 0.01, ***q < 0.001, ****q < 0.0001.

To evaluate the impact of the strains on the gut microbiota composition, we performed 16S rRNA gene analysis on fecal samples from pre-, during and post-treatment days. Before treatment, an average of 228 ± 15 ASVs was detected per mouse and no differences in alpha diversity were observed among the groups. The introduction of streptomycin and DSS decreased the observed richness by 75%, in non-colitic mice, and 55% in all DSS-treated mice from Day 2. All colitic mice exhibited a significantly higher observed richness than the non-colitic and no significant differences were observed between the colitic groups (Fig. 5A). Two days after DSS cessation (Day 10) all groups had similar observed richness (mean = 87 ± 6 ASVs). In terms of beta diversity, group as a variable explains most of the variation observed among all the groups. Depending on the day it explains 18.4 to 30.9% and 11.2 to 39.2% of the variation based on weighted UniFrac distances and Bray–Curtis dissimilarities respectively (Supplementary Table S2). However, the group variable did not significantly explain the variation observed among the colitic groups (Supplementary Table S3, Supplementary Fig. S6) and thus the differences observed across groups were largely explained by the DSS-treatment. The microbiota genus composition of all experimental groups on selected days are depicted in Fig. 5(B).

To further assess the AMT’s impact, we used LIMMA to determine differentially abundant phyla and genera among the colitic groups, including information about sampling day and individual animals. In the phylum level, Proteobacteria (q = 0.002) and Verrucomicrobia (q = 0.002) abundance was significantly elevated on days 8 and 10, in the AMT-receiving groups (data not shown). From the 129 genera detected in total, six were significantly different in their relative abundance, namely *Escherichia/Shigella* (q < 0.0001), *Adlercreutzia* (q = 0.006), *Enteroscipio* (q = 0.006), unclassified *Eggerthellaceae* (q = 0.006), *Enterococcus* (q = 0.01) and *Acinetobacter* (q = 0.02). From these genera, *Escherichia/Shigella*, *Adlercreutzia*, *Enteroscipio* and unclassified *Eggerthellaceae* were consistently more abundant in AMT treated animals (Supplementary Table S4).

## Discussion

Probiotics and/or FMT are agents capable of managing symptoms of UC and induce disease remission in both rodents^56,57^ and humans^58,59^. However, studies with special focus on metabolic products that drive these effects and their mechanisms only now start to rise^60–62^. In this study, we opted to examine EcN *aldh*, an AMT that produces ILA, a natural gut microbial metabolite, on its potential in mitigating symptoms of intestinal inflammation. Overall, the data indicate no or minor differences in acute disease severity (body weight, colon length, histology) among DSS, EcN and EcN *aldh* mice. However, the EcN *aldh* mice showed signs of better initial recovery in terms of lower fecal lcn-2 and altered colon tissue gene expression already three days after DSS cessation. Analysis of expression levels of intestinal epithelial health markers suggested a putative positive effect of the AMT in the initial recovery phase.

DSS is widely used in experimental colitis models and a concentration of 4.5% falls within the high range of doses^63,64^, thus leading to the consistent colitic symptoms that we observed in the animals (Fig. 1B–D). Our results are however somewhat contrasting recent studies, which demonstrate that ILA and ILA-producing strains significantly attenuate weight loss, colon shortening, spleen enlargement and histopathological damage in colitis models^65,66^. Also, the anticipated healing effect of EcN in murine DSS-colitis^67–69^ was not reproduced in the present work. Since a DSS concentration of 3% w/v was used in all the aforementioned studies, we speculate that the high DSS concentration might have increased the severity of the symptoms, possibly masking the full therapeutic potential of the tested strains. Additionally, the efficacy of the ILA-producing strains against colitis is dependent on administration time^65^, thus strain administration before the DSS treatment may have resulted in stronger anti-colitic effect of the AMTs. The streptomycin-induced dysbiosis may have further contributed to symptom severity^40,70^ and the incapability of the interventions to exert their full effect.

Fecal lcn-2 measurements provided evidence that the ILA-producing EcN *aldh* might alleviate the inflamed gut (Fig. 1E), as the animals receiving the strain differed distinctively in their lcn-2 levels. Histopathological damage analysis revealed a weak positive trend of EcN *aldh* on the intestinal epithelium (Fig. 2A), although the disease features were similar among the colitic mice (Fig. 1B–E).

Both resistant strains colonized successfully in the streptomycin-treated mouse model. Their high presence in the ileum may be an indicator of the severity of the colitic symptoms, as degeneration of the ileocecal valve can lead to overgrowth of bacteria in the ileum^71^ and EcN and EcN *aldh* has not been previously observed in the ileum of healthy streptomycin-treated mice^30^. Despite the colonization level, EcN *aldh* did not result in differences between absolute levels of ILA in the feces. That is in contrast to our previous results, where comparable colonization levels in healthy mice led to a significant increase of ILA in fecal matter^30^. It is probable that the concomitant occurrence of streptomycin^40^, DSS^72^, the AMTs, an active inflammation^73,74^, and naturally occurring individualization of the mice microbiota due to cage-effects^75^, increased the inter-individual variability among the mice and, consequently, obscured the effect of EcN *aldh* regarding the amount of fecal ILA. Nevertheless, the ILA was significantly increased in the serum of EcN *aldh* colonized animals as compared to the EcN group (Fig. 3D) indicating that the produced ILA was readily absorbed.

Our RT-qPCR results suggest a better recovery of the EcN *aldh* mice and indicate potential routes through which ILA may function. Specifically, EcN *adlh* colonization led to upregulated the expression of TFF3 (Fig. 4A), a peptide capable of colitis symptoms amelioration^19,76^. It is specifically its luminal administration and not its systemic administration, that has been shown to significantly improve colitic symptoms in murine models^76^, thus rendering EcN *aldh* and its localized effect on TFF3 a promising result that warrants further investigation. Occludin and Muc3 expression was upregulated as well (Fig. 4B and C), although the lack of significant differences between EcN and EcN *aldh* groups suggests the presence of EcN as the effector of that upregulation. It is likely, that EcN *aldh* provides a combinatorial effect of ILA’s and EcN properties.

EcN *aldh* reduced the expression of the pro-inflammatory IL-1β in the colitic mice. That is in accordance with previously reported effects^25,77^. Likewise, lower levels of intestinal tissue expression of Foxp3 were observed in the EcN *aldh* mice (Fig. 4G), an effect that have been reported as a remission response to anti-colitic drug in UC patients^50^. Those results are in line with the observed reduced fecal lcn-2 in the EcN *aldh* mice on day 10 (Fig. 1E).

Most compellingly, the administration of EcN *aldh* led to the downregulation of ICAM1 and iNOS expression (Fig. 4E and F). ICAM1 is thought to have a central role in IBD conditions and treatment with anti-ICAM1 antibodies reduces colitis symptoms in rodents^78,79^. iNOS is negatively associated with intestinal inflammation, and its suppression leads to better outcomes in murine colitis as well. Hence, both proteins are candidate targets for the development of biologic agents^80–83^. Consequently, besides its potential assistance in epithelial recovery (Fig. 4A–C), our results indicate that EcN *aldh* is an interesting candidate to investigate further, as its colonization in the colitic gut delivers results that resemble candidate biologics’ effects.

The AMTs did not influence the alpha diversity, but elicited compositional changes in the gut microbiome, in agreement with previous reports on EcN impact on the microbiota^84^. Streptomycin and DSS affected the observed richness greatly. While their effect was anticipated^40,85^, the groups that were treated with both streptomycin and DSS showed an increase in their alpha diversity when compared to the streptomycin-treated group (Fig. 5A). As DSS is not known to affect intestinal microbiota *in vitro*^86^ and its suspected mechanism in vivo does not involve a direct impact on the microbiota^63^, this effect could be attributed to the host’s immune response, that was triggered by DSS, shaping the microbiome differently.

Even though ILA is known to influence the microbiome composition^66^, we found no major differences between the EcN and the EcN *aldh* groups (Fig. 5). Among the positively influenced genera by both AMT strains, *Eggerthelaceae* has been proposed as a potentially new source of probiotic species^87^. Members of this genus produce ellagic acid metabolites^88^ that can ameliorate colitis symptoms through downregulation of iNOS^89^. Genus *Adlercrutzia* has been also associated with better intestinal health^90,91^, overall suggesting a putative positive influence of EcN on the microbiome. However, the associations among EcN and the aforementioned genera should be treated with care, as the presence of streptomycin may have caused alterations and perturbations that influenced EcN’s effect*.*

EcN and EcN *aldh* did not affect hallmark measures of acute colitis*, i.e.* body weight, colon length, and tissue histology compared to DSS animals. Cui and colleagues revealed that strain administration several days before the DSS treatment is more effective against the colitic symptoms compared to concurrent strain and DSS administration^65^. Consequently, we speculate that a lower DSS dose or a chronic colitis model and an earlier administration of the strains might improve their impact on intestinal health. Studies on cell lines and/or organoids could more closely explore ILA’s effect on the intestine, and studies with animal models should be conducted to further investigate its therapeutic effect. Regarding the safety of the increased ILA systemic distribution, although a previous study of the strain in a conventional mouse model showed no major toxicity signs, such as weight loss, lethargy, changes in fur, eyes, behaviour patterns, diarrhea, etc.^30^, thorough in vitro and in vivo examinations must be conducted to assess the strain’s safety.

Altogether, this study presents an ILA-producing AMT that, when administered in DSS-treated rodents, shows limited therapeutic effect on chemically induced acute colitis, but beneficially affected gene expression markers of epithelial recovery and inflammation in colonic tissue in the initial recovery phase 3 days after treatment cessation. This finding suggests that EcN *aldh* and ILA may assist recovery from conditions that involve intestinal inflammation, however further studies are needed to confirm this.

## Materials and methods

### Strains and bacterial cultures

The engineered probiotic strains used in this study, namely EcN and EcN *aldh*, are modified versions of the wild type *E. coli* Nissle 1917 (tradename Mutaflor, Ardeypharm, Germany) that contain a vector with (EcN *aldh*) or without (EcN) the aromatic lactic acid dehydrogenase (*aldh*, GenBank accession no. WP_014484799.1) from *Bifidobacterium longum* subsp. i*nfantis*^30^. Both strains grew in LB medium supplemented with either 50 µg/ml streptomycin (EcN), or 50 µg/ml streptomycin and 30 µg/ml kanamycin (EcN *aldh*), and incubated at 37 °C at 200 rpm.

Bacterial suspensions of the strains were prepared from overnight cultures, harvested by centrifugation (4000 g for 10 min), washed twice with, and re-suspended in sterile PBS. The concentrations were quantified by flow cytometric detection of GFP on a flow cytometer (MACSQuant® VYB, Miltenyi Biotec) and adjusted to 10^8^ cells/ml. The solutions were kept at room temperature until they were orally administered to the mice.

### Statements of animal care, use and reporting

All methods were performed in accordance with the relevant guidelines and regulations. The Danish Animal Experiments Inspectorate provided ethical approval on the experimental design (Fig. 1A) and protocol, with authorization number: 2020-15-0201-00484 C1. The experiment was carried out at the DTU National Food Institute facilities and the in-house Animal Welfare Committee for animal care and use oversaw the experiments. The animal study was reported in accordance with the ARRIVE guidelines (https://arriveguidelines.org/).

### In vivo experiment

Seven-week-old C57BL/6NTac mice (female, n = 40, Taconic Biosciences, Lille Skensved, Denmark) acclimatized in groups of 10 (Type III cages) for 4 days before the start of the experiment. On day -5, the mice were split into four groups by stratified randomization based on weight, moved to Type II cages and single-housed for the rest of the experiment. No a priori sample size calculations were performed. The groups were: (1) the control group of healthy mice (non-colitic), (2) the disease control mice (DSS), (3) the colitic mice that received EcN (EcN), and (4) the colitic mice that received EcN *aldh* (EcN *aldh*). After 5 days of acclimatization in their single-housing conditions, on Day 0, the appropriate treatment was administered to each group. As both EcN and EcN aldh encode for streptomycin resistance, the chronic colonization model^40^ was used in order to ensure the AMTs’ steady colonization. Accordingly, streptomycin was continuously provided as a drinking solution of streptomycin sulfate (5 g/l) in all groups. Groups DSS, EcN and EcN *aldh* received drinking solutions that additionally contained 4.5% Dextran sulfate sodium (DSS) 40 kDa (Sigma-Aldrich, USA) for the UC-introduction phase from Day 1 to Day 7. All solutions were sterile filtered (Bottle Top Vacuum Filter with 0.22 μm pores). The mice were monitored daily. Each animal was weighted individually, food and fluid intake were registered, and the feces were inspected for signs of disease (stool consistency and blood occurrence). Disease activity index (DAI) was calculated from the registered data according to Mu and colleagues^92^.

Throughout the whole experiment, environmental conditions were kept at 12-h light/ dark cycles, at 22 °C, humidity of 55%, and 50 air changes per hour. The mice had *ab libitum* access to water and feed (SAFE Scientific Diet A30, SAFE, Augy, France). The cages contained aspen bedding (Tapvei Aspen Bedding 4HBB), nesting material (EnviroDri), a rodent stick (Tapvei S-Bricks Aspen) and a hide (Mouse House, red polycarbonate).

On Day 4, all groups were orally gavaged with 100 µl of either bacterial solution (10^8^ cells/ml) of EcN or EcN *aldh* (EcN and EcN *aldh* groups), or with sterile PBS (non-colitic and DSS groups). On Day 7, DSS treatment was ceased, followed 3 days post-treatment. On Day 7, three animals needed to be sacrificed due to the established human-endpoint criteria, which correspond to weight loss below the 20%. Data from those mice, one from the EcN and two from the EcN *aldh* group, were excluded from downstream analysis. On Day 10, the remaining mice were sedated by hypnorm/midazolam (0.1 ml/10 g SC) and euthanized by cervical dislocation.

### Collection of in vivo samples

Two fecal pellets from each mouse were collected daily and stored at -80 °C until further processing. Peripheral blood samples were collected on Day -1, Day 3, and Day 9. All blood samples were processed the day of collection; they were incubated at room temperature for 30 to 60 min, to allow coagulation, and then centrifuged for 10 min at 2000 rpm followed by supernatant aspiration to obtain the serum.

Post-termination, the colon was removed, and its length was measured. Gut content was collected from ileum, cecum and colon for plating and CFU counting. Two colonic tissue samples (approx. 0.5 cm each) were collected and either stored in RNA later (Invitrogen, USA) in -20 °C, for subsequent RNA extraction, or fixed and embedded in paraffin for histological analysis.

### Lipocalin-2 detection assays

Frozen serum samples were thawed at 4 °C for 30 min and then allowed to equilibrate to room temperature before processing. Frozen fecal samples were thawed at room temperature and reconstituted in PBS. Lcn-2 was quantified using Lipocalin-2 (NGAL) Mouse ELISA Kit (Abcam, UK) according to the manufacturer’s instructions. The broad and narrow spectrum versions of the kit were used for the fecal and serum samples respectively. Measurements were conducted with the BioTek ELx800 Absorbance Microplate Reader (BioTek, USA).

### Histology analysis

Five-µm sections were cut (Epredia HM 450 Sliding Microtome, Thermo Fisher Scientific, US) from the paraffin-embedded tissue and stained with hematoxylin (Avantor, USA) and eosin (0.5% solution, Sigma-Aldrich, USA). Histopathological damage was evaluated by two investigators in a blinded manner (Supplementary Table S5), by light microscopy regarding (1) the severity and extent of the inflammatory cell infiltration, and (2) the changes of epithelial and mucosal architecture, using the criteria of Erben and colleagues^93^.

### Gut content samples—Plate CFU counting

The gut content samples were placed at 4 °C, until processing. On the same day, the samples were diluted 1:5 w/v in sterile MilliQ (MQ) water and vortexed in 30-s intervals, until fully dissolved. Ten-fold dilutions were prepared and plated, for plate counting in triplicates^94^, on LB plates with either 50 µg/ml streptomycin (selects for both AMTs), or 30 µg/ml kanamycin and 50 µg/ml streptomycin (EcN *aldh* selection).

### Fecal DNA isolation

The fecal samples were thawed at 4 °C for approximately 30 min. DNA was extracted using the DNeasy PowerSoil Kit (12,855-100, Qiagen, Netherlands) modified on step 5 to include a two-step centrifugation: first 3 min at 13,000 rpm, and, after removing most of the supernatant, for 1 min at 13,000 rpm. DNA concentrations were measured with the Qubit dsDNA HS and Qubit dsDNA BS (when appropriate) assay kits (Thermo Fisher Scientific, USA). The DNA was stored at -20 °C until further use.

### LC-HRMS analysis—aromatic amino acids and metabolites profiling

The fecal samples were thawed at 4 °C for approximately 30 min. The thawed pellets were diluted 1:5 w/v in sterile MQ water, and the samples were vortexed in 30-s intervals, until fully dissolved, and then processed as previously described^30^. Briefly, a liquid–liquid extraction was employed, acetonitrile was used as a protein precipitation agent, and the samples underwent a series of centrifugations to extract the analytes, then they were concentrated to dryness, and diluted before injection in the column. Internal standard (IS) solutions were prepared for three stable isotopes, namely L-tryptophan (indole-d5), L-phenylalanine (ring-d5) and L-tyrosine (ring-d4), in MQ water (16 µg/ml). Standard solutions (SDs) were prepared for the analytes (the three aromatic amino acids and their respective lactic acid and pyruvic acid derivatives) and the three stable isotopes in their respective diluents to a final concentration of 1 mg/ml (Supplementary Table S6). The SDs were further diluted to 0.1 µg/ml, 0.5 µg/ml, 1 µg/ml, 2 µg/ml, 4 µg/ml, 10 µg/ml and 20 µg/ml and the IS diluted to 4 µg/ml. A calibration curve was calculated by the analysis of the SDs. All serum samples were processed according to Zhu and colleagues^95^.

### RNA extraction and RT-qPCR

RNA was extracted from colonic tissue samples, stored in RNAlater, using the RNAqueous™-4PCR Total RNA Isolation Kit (Invitrogen, USA), according to manufacturer’s instructions. The RNA yield was measured with the Qubit HS RNA assay (Invitrogen, USA) and the samples were stored in − 80 °C. The RNA was reverse transcribed using the SuperScript™ IV VILO™ Master Mix (Invitrogen, USA). An ezDNase (Invitrogen, USA) treatment step preceded the reverse transcription reaction. Both steps were performed according to the kit protocols, with the reverse transcription reactions scaled up to 40 µl.

Relative quantification analysis of the genes-of-interest expression levels was performed with mRNA-specific primers (Supplementary Table S7) using the cDNA. The LightCycler 480 SYBR Green I Master Mix (Roche, Switzerland) was used for the assay according to its protocol, with a final volume of 10 µl. All samples were run in triplicates on the LightCycler 480 Instrument II (Roche, Switzerland). Single outliers were excluded from average Ct-value calculation in case the triplicates showed SD_Ct_ values > 0.5. Non-POU Domain Containing Octamer Binding protein (NONO) served as a reference gene^96^. Data processing was performed with the LinRegPCR software^97^. The results were analyzed with the ΔΔCT method^98^ using the healthy control group as reference.

### 16S rRNA amplicon sequencing

16S rRNA gene sequencing was performed on the Ion Torrent platform on the fecal samples from Days -1, 6, 8, and 10. The 16S rRNA gene V3-region was PCR amplified using the Phusion High-Fidelity DNA Polymerase (Thermo Fisher Scientific, USA), according to the manufacturer’s instructions, with a 20 µl total reaction volume. We used forward primer: PBU; 5ʹ-A-adapter-TCAG-barcode-CCTACGGGAGGCAGCAG-3ʹ and reverse primer PBR_P1: 5ʹ-trP1-adapter-ATTACCGCGGCTGCTGG-3ʹ. PBUs contained a unique barcode of 10–12 bp (Ion Xpress™ Barcode Adapters) for each sample and PBR_P1 the universal trP1 adapter, both needed for Ion Torrent sequencing. The PCR program comprised denaturation (98 °C for 30 secs), 24 cycles amplification (98 °C for 15 s, 72 °C for 30 s), and finally 72 °C for 5 min. PCR products were purified with the HighPrep PCR clean-up system (MagBIO), according to the manufacturer. DNA concentrations were measured with the Qubit dsDNA HS assay (Invitrogen, USA) and samples were pooled to obtain equimolar libraries. Sequencing was performed on the Ion S5™ sequencing system, on two separate Ion 520™ chips prepared with the Ion OneTouch™ 2 system.

### Microbial community analysis—bioinformatic and statistical analysis

16S rRNA gene amplicon reads were processed using an in-house pipeline^99^. Raw amplicon sequences were demultiplexed by cutadapt (v. 4.1)^100^ and denoised using DADA2 (v. 1.22)^101^. ASVs were classified against rdp_train_set_18^102^. All downstream processing was done by Phyloseq (v.1.42.0) running in R (v. 4.2)^103^ and ASV decontamination was conducted with the decontam package^104^.

### Statistical analysis

Except for the 16S rRNA analysis, all statistical analysis was performed in GraphPad Prism, version 9 (GraphPad Software Inc., USA). Normal distribution of the variables was assessed with the Kolmogorov–Smirnov test (p > 0.05). Outliers were identified and removed using the ROUT method (Q = 1%). For LC-HRMS data analysis, values below the limit of quantification (LOQ) and above the limit of detection were replaced with the LOQ/2. A p value of less than 0.05 was considered significant. The three prematurely terminated mice were removed from the analysis, hence, data presented in this study comprises results from n = 10 (Non-colitic group), n = 10 (DSS group), n = 9 (EcN group), n = 8 (EcN *aldh* group) mice. However, due to missing fecal samples fewer samples were processed on LC-HRMS, for 16S rRNA gene amplicon sequencing and for fecal lcn-2 measurements (Supplementary Table S1).

Alpha diversity was analysed by paired mixed effects model (REML), followed by Tukey multiple comparison test to correct for multiple testing. Beta-diversity contributing factors were analysed by permutational analysis of variance (PERMANOVA)^105^. Differential abundance analysis was conducted with the Limma model^106^. A Spearman’s correlation analysis was conducted to study the relationship of the differentially abundant genera, as detected in each group, with collection day, fecal lcn-2 levels and the aromatic lactic acids (aLAs) and aromatic amino acids (AAs) concentration in the fecal samples. To correct for multiple testing, the Benjamini–Hochberg method was applied^107^ and all q values presented stand for the Benjamini–Hochberg adjusted p-value. All analyses were conducted in R^103^ and bar and box plots were created in GraphPad Prism, version 9 (GraphPad Software Inc., USA).

### Supplementary Information

Supplementary Information.

## Data Availability

Data available from the corresponding author (M.F.L.) upon request.
