# Supplementary material for: Potential of using an engineered indole lactic acid producing Escherichia coli Nissle 1917 in a murine model of colitis
Source: Sci Rep. 2024 Jul 30;14:17542. doi: 10.1038/s41598-024-68412-9 (PMC11289411; doi:10.1038/s41598-024-68412-9)
Supplement: Supplementary file 1 — Supplementary Information. [file 41598_2024_68412_MOESM1_ESM.docx]

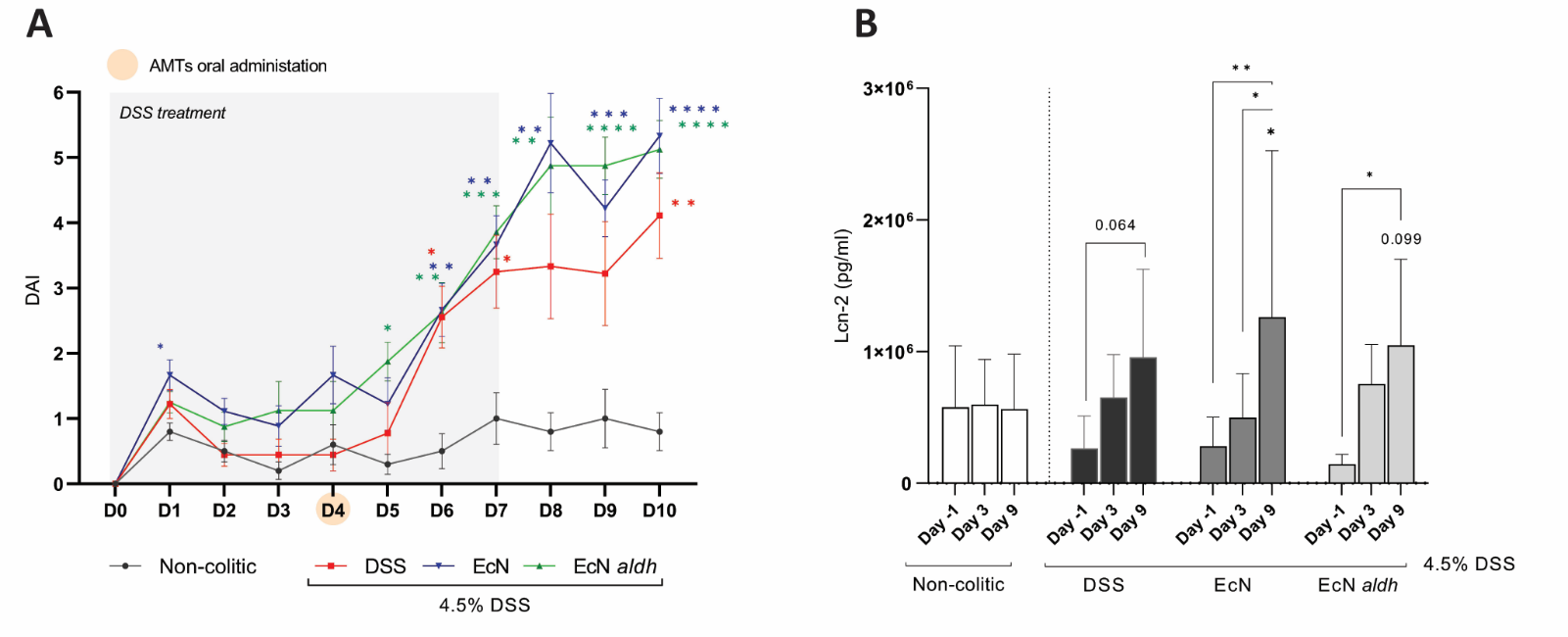
**Supplementary Figure S1: DSS-induced colitis: markers of disease** (A) DAI calculations of all animal groups. The dots depict the mean ± SEM of the biological replicates. Statistical significance was calculated by paired comparisons using the REML model, Tukey post-hoc correction, * q < 0.05, ** q < 0.01, *** q < 0.001, **** q < 0.0001 (B) Serum lcn-2 measurements on selective days of the experiment. Statistical analysis was performed with the mixed effects model (REML), uncorrected Fisher’s LSD, * p < 0.05, ** p < 0.01, *** p < 0.001, **** p < 0.0001


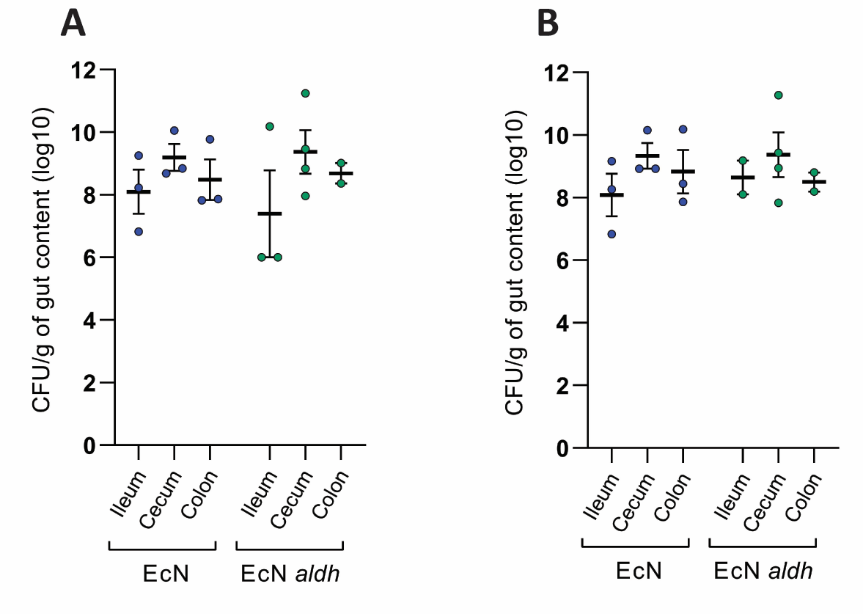


**Supplementary Figure S2. Colonization levels of the administered AMT strains.** CFU counts of EcN and EcN *aldh* in the gut content of the treated animals on the last day of the experiment (D10). The platting conducted on (A) streptomycin and kanamycin selective plates that select for the strain and the plasmid vector and (B) streptomycin selective plates that select only for the strain. The graphs represent the mean ± SEM from 3 independent biological replicates. Unpaired t-tests with Welch correction confirmed the lack of significant differences in the distribution of each strain among the different compartments. Unpaired Mann-Whitney tests confirmed the absence of significant plasmid loss (comparisons between the CFU counts from the streptomycin and kanamycin selective plates and the CFU counts from the streptomycin selective plates)


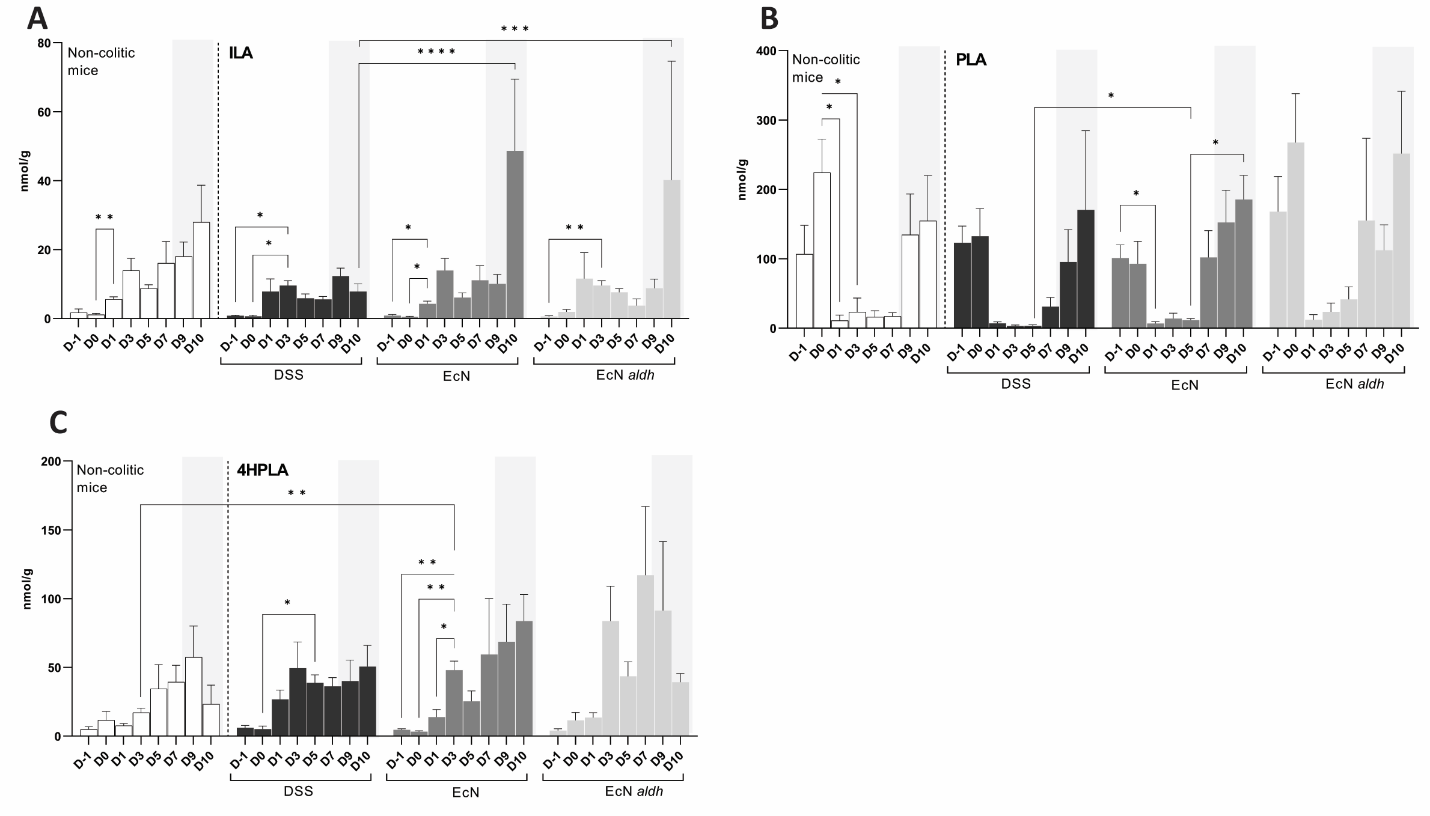


**Supplementary Figure S3: Impact of the AMT strains on fecal concentration of the aLAs.** Concentration of (A) ILA, (B) PLA and (C) 4HPLA in fecal samples of the mice on selected days. The bars depict the mean ± SEM of the biological replicates. The REML model, Tukey test correction, determined statistical significance. * q < 0.05, ** q < 0.01, *** q < 0.001, **** q < 0.0001


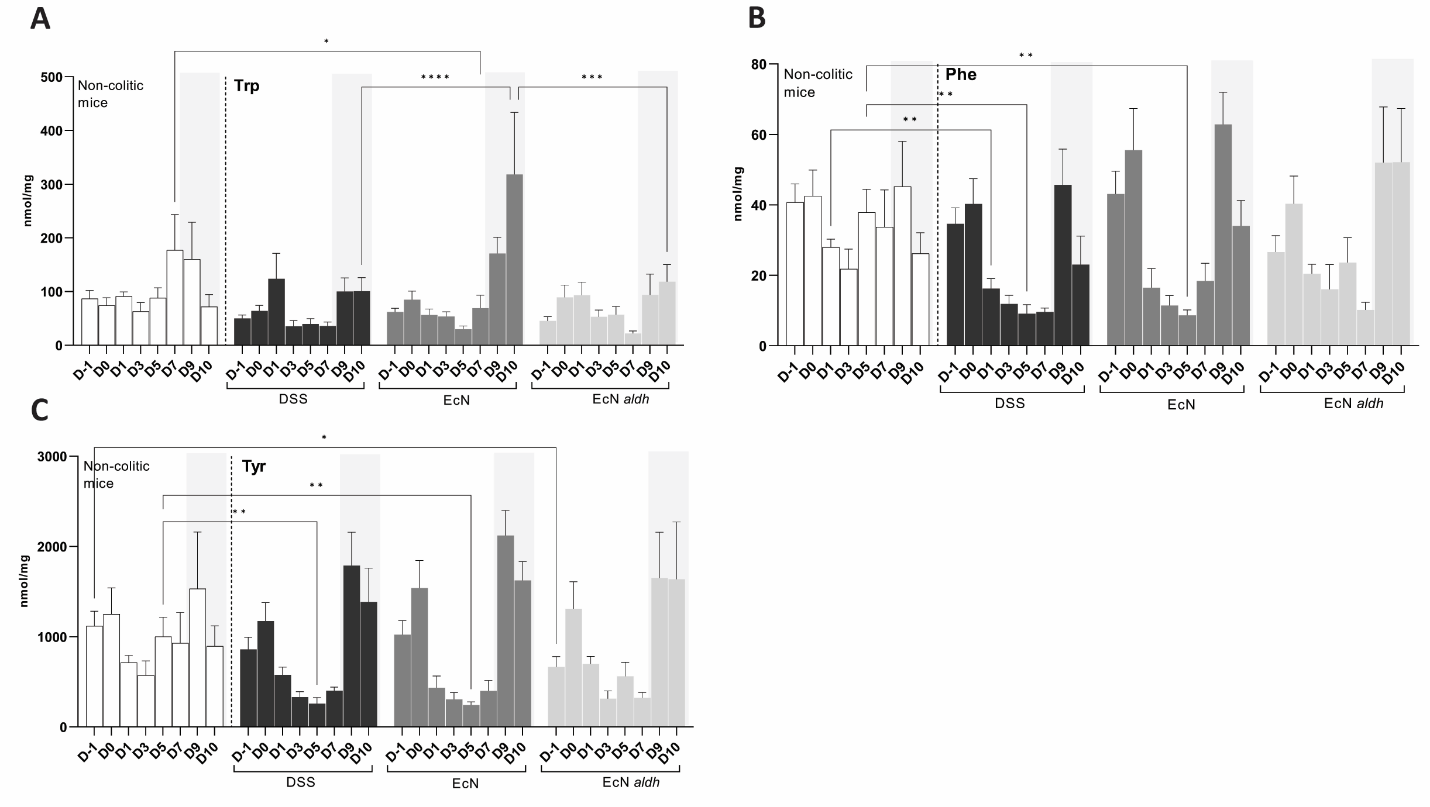


**Supplementary Figure S4: Impact of the AMT strains on fecal concentration of the aromatic amino acids.** Concentration of (A) tryptophan (Trp), (B) phenylalanine (Phe) and (C) tyrosine (Tyr) in fecal samples of the mice on selected days. The bars depict the mean ± SEM. The REML model, Fisher’s LSD test, determined statistical significance. * p < 0.05, ** p < 0.01, *** p < 0.001, **** p < 0.0001


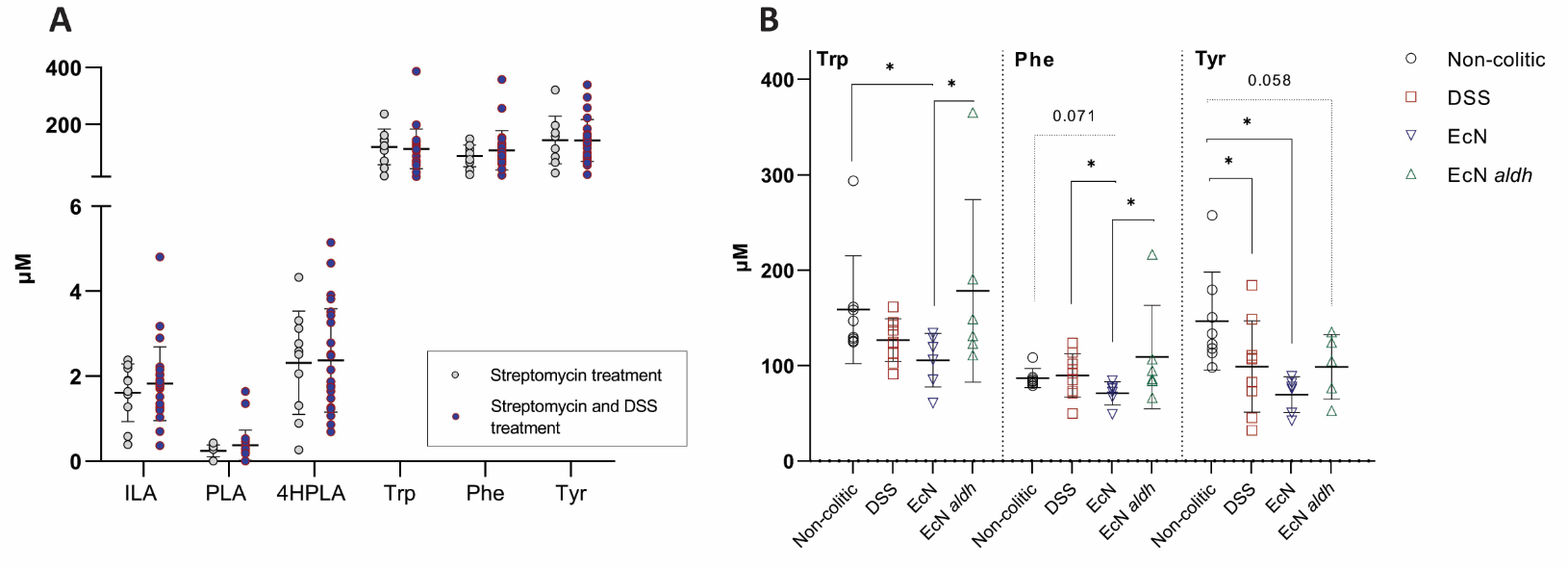
**Supplementary Figure S5: aLAs and AAs in the serum of the mice.** A. Concentration of the aLAs and AAs in serum samples on Day 3. B. AAs concentrations in serum samples on Day 9. The graphs illustrate the mean ± SD of the biological replicates (n = 8, 9, 7, 6 in the non-colitic, DSS, EcN and EcN aldh groups respectively). Each symbol represents an individual mouse. Statistical significance was calculated with the Kruskal-Wallis method, uncorrected Dunn’s test and one-way ANOVA, uncorrected Dunns test (Tyrosine concentration samples) * p < 0.05, ** p < 0.01, *** p < 0.001


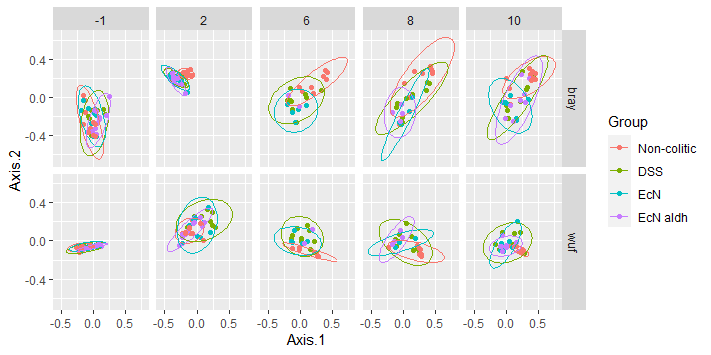


**Supplementary Figure S6: Beta diversity of the experimental groups.** PCoA plots using Brey-Curtis dissimilarity index (bray) and weighted UniFrac distances (wuf) of the ASV composition in the four experimental groups.

**Supplementary Table S1:** Number of samples processed in LC-HRMS and 16S amplicon analysis

| **Group name** | **LC-HRMS analysis** | | | | | | | |
| --- | --- | --- | --- | --- | --- | --- | --- | --- |
|  | **D-1** | **D0** | **D3** | **D5** | | **D7** | **D9** | **D10** |
| **Non-colitic** | 8 | 10 | 10 | 10 | | 8 | 9 | 7 |
| **DSS** | 10 | 10 | 5 | 7 | | 7 | 9 | 9 |
| **EcN** | 8 | 9 | 10 | 9 | | 6 | 8 | 7 |
| **EcN aldh** | 10 | 10 | 8 | 8 | | 5 | 6 | 4 |
| **Group name** | **16S amplicon analysis** | | | | | |  |  |
|  | **D-1** | **D2** | **D6** | **D8** | | **D10** |  |  |
| **Non-colitic** | 10 | 10 | 8 | 9 | | 10 |  |  |
| **DSS** | 10 | 9 | 10 | 9 | | 10 |  |  |
| **EcN** | 9 | 9 | 5 | 5 | | 8 |  |  |
| **EcN aldh** | 8 | 8 | 4 | 4 | | 8 |  |  |
| **Group name** | **Fecal Lcn-2 analysis** | | | |  |  |  |  |
|  | **D0** | **D4** | **D10** |  |  |  |  |  |
| **Non-colitic** | 10 | 9 | 9 |  |  |  |  |  |
| **DSS** | 10 | 10 | 7 |  |  |  |  |  |
| **EcN** | 8 | 8 | 7 |  |  |  |  |  |
| **EcN aldh** | 8 | 7 | 7 |  |  |  |  |  |

**Supplementary Table S2:** Permutational multivariate analysis of variance (PERMANOVA) results calculated from weighted UniFrac distances and Bray-Curtis dissimilarities for samples from all mice groups on all collection days

|  | Collection Day | Sum of Squares | R2 | P value |
| --- | --- | --- | --- | --- |
| Analysis of weighted UniFrac distance | Day -1 | 0.7573 | 0.18494 | 0.018 |
|  | Day 2 | 11.545 | 0.30607 | 0.001 |
|  | Day 6 | 0.71769 | 0.30978 | 0.004 |
|  | Day 8 | 0.69467 | 0.2707 | 0.033 |
|  | Day 10 | 0.74769 | 0.29107 | 0.001 |
|  | **Collection Day** | **Sum of Squares** | **R2** | **P value** |
| Analysis of Bray-Curtis dissimilarity | Day -1 | 0.8951 | 0.1117 | 0.052 |
|  | Day 2 | 40.485 | 0.39245 | 0.001 |
|  | Day 6 | 21.684 | 0.31445 | 0.001 |
|  | Day 8 | 17.758 | 0.25505 | 0.002 |
|  | Day 10 | 13.744 | 0.15824 | 0.005 |

**Supplementary Table S3:** Permutational multivariate analysis of variance (PERMANOVA) results calculated from weighted UniFrac distances and Bray-Curtis dissimilarities for the colitic groups on days after DSS treatment initiation

|  | Collection Day | Sum of Squares | R2 | P value |
| --- | --- | --- | --- | --- |
| Analysis of weighted UniFrac distance | Day 2 | 0.39059 | 0.15908 | 0.059 |
|  | Day 6 | 0.069475 | 0.034738 | 0.001051 |
|  | Day 8 | 0.18254 | 0.16413 | 0.249 |
|  | Day 10 | 0.08272 | 0.05073 | 0.807 |
|  | **Collection Day** | **Sum of Squares** | **R2** | **P value** |
| Analysis of Bray-Curtis dissimilarity | Day 2 | 0.5056 | 0.08964 | 0.172 |
|  | Day 6 | 0.5318 | 0.15986 | 0.186 |
|  | Day 8 | 0.4836 | 0.13803 | 0.284 |
|  | Day 10 | 0.4494 | 0.07684 | 0.663 |

**Supplementary Table S4:** Relative abundance for the top 20 most abundant genera per group and per collection day. *Non col.: non colitic group*

| **Day 8** | **EcN *aldh*** | 4.46 | 8.18 | 10.12 | 10.18 | 24.33 | 0.90 | 3.08 | 3.35 | 0.03 | 7.07 | 0.12 | 0.94 | 0.28 | 5.16 | 0.80 | 0.83 | 9.04 | 0.74 | 2.40 | 5.55 | 2.44 |
| --- | --- | --- | --- | --- | --- | --- | --- | --- | --- | --- | --- | --- | --- | --- | --- | --- | --- | --- | --- | --- | --- | --- |
|  | **EcN** | 6.97 | 6.98 | 9.38 | 19.13 | 27.62 | 2.23 | 2.98 | 3.96 | 0.00 | 0.25 | 0.06 | 0.99 | 0.55 | 6.12 | 1.53 | 1.41 | 5.45 | 1.44 | 0.80 | 1.18 | 0.97 |
|  | **DSS** | 3.27 | 3.68 | 11.89 | 18.10 | 19.32 | 4.35 | 2.33 | 2.82 | 0.01 | 0.80 | 0.39 | 0.70 | 1.99 | 15.04 | 0.72 | 0.95 | 4.40 | 1.93 | 1.91 | 1.78 | 3.63 |
|  | **Non col.** | 0.20 | 4.44 | 4.20 | 33.24 | 8.10 | 7.05 | 1.74 | 0.96 | 0.02 | 0.02 | 1.00 | 2.35 | 0.90 | 20.23 | 0.32 | 0.34 | 3.99 | 0.04 | 9.96 | 0.05 | 0.86 |
| **Day 6** | **EcN *aldh*** | 0.55 | 1.42 | 15.81 | 22.76 | 7.41 | 2.74 | 4.72 | 2.21 | 0.00 | 0.88 | 0.00 | 0.80 | 0.39 | 13.93 | 0.21 | 1.66 | 8.39 | 1.05 | 1.91 | 5.15 | 7.98 |
|  | **EcN** | 1.55 | 8.64 | 13.88 | 14.69 | 23.98 | 1.75 | 1.74 | 3.69 | 0.00 | 0.54 | 0.65 | 1.28 | 0.56 | 6.16 | 4.04 | 1.35 | 4.38 | 0.75 | 5.70 | 3.92 | 0.76 |
|  | **DSS** | 3.13 | 4.13 | 19.10 | 14.89 | 18.58 | 3.36 | 2.19 | 1.81 | 0.04 | 0.67 | 0.09 | 0.57 | 1.51 | 9.62 | 0.70 | 1.55 | 5.67 | 1.37 | 4.96 | 3.37 | 2.68 |
|  | **Non col.** | 0.13 | 1.69 | 1.29 | 27.44 | 5.44 | 7.76 | 0.93 | 0.13 | 0.15 | 0.13 | 0.62 | 0.34 | 14.93 | 26.11 | 1.37 | 0.06 | 4.14 | 0.00 | 5.46 | 0.01 | 1.86 |
| **Day 2** | **EcN *aldh*** | 1.26 | 0.31 | 18.51 | 3.48 | 7.13 | 0.49 | 3.07 | 0.73 | 0.89 | 15.00 | 13.90 | 5.99 | 1.31 | 0.93 | 0.13 | 1.12 | 6.11 | 3.30 | 6.45 | 9.11 | 0.78 |
|  | **EcN** | 2.18 | 0.18 | 24.00 | 5.25 | 3.17 | 1.36 | 1.61 | 0.48 | 0.28 | 5.20 | 17.17 | 2.78 | 0.07 | 4.01 | 0.27 | 1.08 | 6.05 | 2.02 | 12.61 | 9.52 | 0.72 |
|  | **DSS** | 1.72 | 0.23 | 34.17 | 10.01 | 0.45 | 0.69 | 2.05 | 0.80 | 0.23 | 3.86 | 8.16 | 1.69 | 0.16 | 1.32 | 0.36 | 0.74 | 7.94 | 3.09 | 7.87 | 14.32 | 0.14 |
|  | **Non col.** | 0.04 | 0.16 | 26.61 | 1.53 | 0.17 | 0.01 | 13.64 | 0.01 | 0.57 | 0.04 | 11.27 | 4.76 | 0.00 | 0.10 | 12.88 | 0.24 | 7.78 | 0.00 | 5.82 | 0.01 | 14.36 |
| **Day -1** | **EcN *aldh*** | 1.97 | 6.61 | 0.66 | 22.06 | 15.20 | 4.91 | 4.09 | 3.32 | 3.04 | 2.90 | 3.04 | 0.26 | 0.00 | 9.79 | 3.71 | 1.19 | 12.33 | 0.02 | 3.97 | 0.34 | 0.57 |
|  | **EcN** | 1.84 | 9.68 | 0.34 | 16.96 | 14.60 | 2.87 | 2.37 | 1.33 | 7.18 | 14.07 | 7.32 | 0.23 | 0.01 | 5.29 | 2.41 | 0.58 | 7.78 | 0.02 | 4.29 | 0.24 | 0.61 |
|  | **DSS** | 1.86 | 14.32 | 0.59 | 13.15 | 16.55 | 2.33 | 4.43 | 2.02 | 5.55 | 9.18 | 4.56 | 0.19 | 0.00 | 8.35 | 3.06 | 1.67 | 8.50 | 0.03 | 2.62 | 0.60 | 0.44 |
|  | **Non col.** | 0.98 | 11.59 | 0.37 | 7.09 | 19.53 | 1.45 | 3.21 | 1.70 | 8.28 | 17.90 | 5.43 | 0.25 | 0.00 | 4.91 | 1.90 | 1.02 | 12.09 | 0.01 | 1.45 | 0.37 | 0.47 |
| **Genus** | | Alistipes | Anaerostipes | Bacteroides | Duncaniella | Family_Lachnospiraceae | Family_Muribaculaceae | Family_Ruminococcaceae | Intestinimonas | Lactobacillus | Ligilactobacillus | Limosilactobacillus | Neglecta | Olsenella | Order_Bacteroidales | Order_Clostridiales | Oscillibacter | Others | Parabacteroides | Paramuribaculum | Phocaeicola | Phylum_Firmicutes |
| **Family** | | Rikenellaceae | Lachnospiraceae | Bacteroidaceae | Muribaculaceae | Lachnospiraceae | Muribaculaceae | Ruminococcaceae | Ruminococcaceae | Lactobacillaceae | Lactobacillaceae | Lactobacillaceae | Ruminococcaceae | Atopobiaceae | Order_Bacteroidales | Order_Clostridiales | Ruminococcaceae | Others | Porphyromonadaceae | Muribaculaceae | Bacteroidaceae | Phylum_Firmicutes |
| **Order** | | Bacteroidales | Clostridiales | Bacteroidales | Bacteroidales | Clostridiales | Bacteroidales | Clostridiales | Clostridiales | Lactobacillales | Lactobacillales | Lactobacillales | Clostridiales | Coriobacteriales | Bacteroidales | Clostridiales | Clostridiales | Others | Bacteroidales | Bacteroidales | Bacteroidales | Phylum_Firmicutes |
| **Class** | | Bacteroidia | Clostridia | Bacteroidia | Bacteroidia | Clostridia | Bacteroidia | Clostridia | Clostridia | Bacilli | Bacilli | Bacilli | Clostridia | Coriobacteriia | Bacteroidia | Clostridia | Clostridia | Others | Bacteroidia | Bacteroidia | Bacteroidia | Phylum_Firmicutes |
| **Phylum** | | Bacteroidetes | Firmicutes | Bacteroidetes | Bacteroidetes | Firmicutes | Bacteroidetes | Firmicutes | Firmicutes | Firmicutes | Firmicutes | Firmicutes | Firmicutes | Actinobacteria | Bacteroidetes | Firmicutes | Firmicutes | Others | Bacteroidetes | Bacteroidetes | Bacteroidetes | Firmicutes |

**Supplementary Table S5:** Scoring scheme for histopathological analysis of chemically-induced colonic inflammation

| *Inflammatory cell infiltrate:* | | *Score 1* | *Intestinal architecture:* | | *Score 2* |
| --- | --- | --- | --- | --- | --- |
| *Severity* | ***Extent*** |  | ***Epithelial changes*** | ***Mucosal architecture*** |  |
| Mild | Mucosa | 1 | Focal erosions |  | 1 |
| Moderate | Mucosa and submucosa | 2 | Erosions | ± Focal ulcerations | 2 |
| Marked | Transmural | 3 |  | Extended ulcerations ± granulation tissue ± pseudopolyps | 3 |

Sum of scores 1 and 2: 0-6

**Supplementary Table S6:** ISs, AA and aLA analytes, their dilutants and their respective internal standards (when applicable)

| Metabolite | Dissolved in | Internal standard |
| --- | --- | --- |
| L-tryptophan (indole-d5) | H_2_O | - |
| L-phenylalanine (ring-d5) | H_2_O | - |
| L-tyrosine (ring-d4) | 1M HCl | - |
| Tryptophan | H_2_O | L-tryptophan (indole-d5) |
| Indolactic acid | H_2_O | L-tryptophan (indole-d5) |
| Indolepyruvic acid | EtOH | L-tryptophan (indole-d5) |
| Phenylalanine | H_2_O | L-phenylalanine (ring-d5) |
| Phenyllactic acid | EtOH | L-phenylalanine (ring-d5) |
| Phenylpyruvic acid | 50% EtOH | L-phenylalanine (ring-d5) |
| Tyrosine | 1M HCl | L-tyrosine (ring-d4) |
| p-hydroxyphenyllactic acid | EtOH | L-tyrosine (ring-d4) |
| 3-(4-hydroxyphenyl)-pyruvic acid | EtOH | L-tyrosine (ring-d4) |

**Supplementary Table S7:** Primers used in the RT-qPCR relative expression analysis

| **Gene** | **Protein** | **Forward sequence (5'-3')** | **Reverse sequence (5'-3')** | **Tm (F/R) °C** |
| --- | --- | --- | --- | --- |
| TFF3 | Trefoil factor 3 | TTGCTGGGTCCTCTGGGATAG | TACACTGCTCCGATGTGACAG | 62.8 / 61.5 |
| OCL | Ocludin | TTGAAAGTCCACCTCCTTACAGA | CCGGATAAAAAGAGTACGCTGG | 60.6 / 60.7 |
| Muc3 | Mucin 3 | GCCGTGAATTGTATGAACGGA | CGCAGTTGACCACGTTGACTA | 60.7 / 62.6 |
| IL-1b | Interleukin 1 beta | GAAATGCCACCTTTTGACAGTG | TGGATGCTCTCATCAGGACAG | 60.2 / 61 |
| ICAM-1 | Intercellular Adhesion Molecule 1/CD54 | GTGATGCTCAGGTATCCATCCA | CACAGTTCTCAAAGCACAGCG | 61.3 / 62 |
| iNOS | Nitric oxide synthase (nos2) | GTTCTCAGCCCAACAATACAAGA | GTGGACGGGTCGATGTCAC | 60.5 / 62.4 |
| Foxp3 | Forkhead box P3/scurfin | ACCATTGGTTTACTCGCATGT | TCCACTCGCACAAAGCACTT | 60 / 62.3 |
| RIPK1 | Receptor-interacting Ser/Thr -protein kinase 1 | GAAGACAGACCTAGACAGCGG | CCAGTAGCTTCACCACTCGAC | 61.6 / 62.1 |
| AhR | Aryl hydrocarbon receptor | ACATACGCCGGTAGGAAGAGA | GGTCCAGCTCTGTATTGAGGC | 62.2 / 62.2 |
| TLR9 | Toll-like receptor 9 | ATGGTTCTCCGTCGAAGGACT | GAGGCTTCAGCTCACAGGG | 62.7 / 62 |
| TGF-b | Transforming growth factor beta | ATGTCACGGTTAGGGGCTC | GGCTTGCATACTGTGCTGTATAG | 61.1 / 61 |
| nono | Non-POU Domain Containing Octamer Binding | ACGAACCCTAGCGGAAATTGC | AGGTTGCGGACTGTAAGGGAT | 63 / 63 |
